# Respiratory Motion-Registered Isotropic Whole-Heart T_2_ Mapping in Patients With Acute Non-ischemic Myocardial Injury

**DOI:** 10.3389/fcvm.2021.712383

**Published:** 2021-09-29

**Authors:** Karolina Dorniak, Lorenzo Di Sopra, Agnieszka Sabisz, Anna Glinska, Christopher W. Roy, Kamil Gorczewski, Davide Piccini, Jérôme Yerly, Hanna Jankowska, Jadwiga Fijałkowska, Edyta Szurowska, Matthias Stuber, Ruud B. van Heeswijk

**Affiliations:** ^1^Department of Noninvasive Cardiac Diagnostics, Medical University of Gdansk, Gdansk, Poland; ^2^Department of Radiology, Lausanne University Hospital (CHUV) and University of Lausanne (UNIL), Lausanne, Switzerland; ^3^Second Department of Radiology, Medical University of Gdansk, Gdansk, Poland; ^4^Siemens Healthineers, Erlangen, Germany; ^5^Advanced Clinical Imaging Technology, Siemens Healthcare AG, Lausanne, Switzerland; ^6^Center for Biomedical Imaging (CIBM), Lausanne, Switzerland

**Keywords:** cardiac magnetic resonance(CMR), acute non-ischemic myocardial injury, isotropic 3D imaging, T_2_ mapping, respiratory motion correction

## Abstract

**Background:** T_2_ mapping is a magnetic resonance imaging technique that can be used to detect myocardial edema and inflammation. However, the focal nature of myocardial inflammation may render conventional 2D approaches suboptimal and make whole-heart isotropic 3D mapping desirable. While self-navigated 3D radial T_2_ mapping has been demonstrated to work well at a magnetic field strength of 3T, it results in too noisy maps at 1.5T. We therefore implemented a novel respiratory motion-resolved compressed-sensing reconstruction in order to improve the 3D T_2_ mapping precision and accuracy at 1.5T, and tested this in a heterogeneous patient cohort.

**Materials and Methods:** Nine healthy volunteers and 25 consecutive patients with suspected acute non-ischemic myocardial injury (sarcoidosis, *n* = 19; systemic sclerosis, *n* = 2; acute graft rejection, *n* = 2, and myocarditis, *n* = 2) were included. The free-breathing T_2_ maps were acquired as three ECG-triggered T_2_-prepared 3D radial volumes. A respiratory motion-resolved reconstruction was followed by image registration of the respiratory states and pixel-wise T_2_ mapping. The resulting 3D maps were compared to routine 2D T_2_ maps. The T_2_ values of segments with and without late gadolinium enhancement (LGE) were compared in patients.

**Results:** In the healthy volunteers, the myocardial T_2_ values obtained with the 2D and 3D techniques were similar (45.8 ± 1.8 vs. 46.8 ± 2.9 ms, respectively; *P* = 0.33). Conversely, in patients, T_2_ values did differ between 2D (46.7 ± 3.6 ms) and 3D techniques (50.1 ± 4.2 ms, *P* = 0.004). Moreover, with the 2D technique, T_2_ values of the LGE-positive segments were similar to those of the LGE-negative segments (T_2LGE−_= 46.2 ± 3.7 vs. T_2LGE+_ = 47.6 ± 4.1 ms; *P* = 0.49), whereas the 3D technique did show a significant difference (T_2LGE−_ = 49.3 ± 6.7 vs. T_2LGE+_ = 52.6 ± 8.7 ms, *P* = 0.006).

**Conclusion:** Respiratory motion-registered 3D radial imaging at 1.5T led to accurate isotropic 3D whole-heart T_2_ maps, both in the healthy volunteers and in a small patient cohort with suspected non-ischemic myocardial injury. Significantly higher T_2_ values were found in patients as compared to controls in 3D but not in 2D, suggestive of the technique's potential to increase the sensitivity of CMR at earlier stages of disease. Further study will be needed to demonstrate its accuracy.

## Background

The T_2_ relaxation time is one of the physiology-dependent properties of a tissue in a magnetic field that governs the image contrast in magnetic resonance imaging (MRI). In the myocardium, it increases in the presence of edema, which makes the T_2_ relaxation time a useful indicator of acute myocardial injury irrespective of its etiology (e.g., inflammatory, toxic, or ischemic) (1). T_2_ mapping, i.e., quantifying the T_2_ relaxation time in every pixel, has therefore seen increased use for the diagnosis of acute myocardial injury in recent years (2). T_2_ mapping has been shown as to be an effective complementary tool in inflammatory diseases such myocarditis (3), systemic sclerosis (4), and sarcoidosis (5).

Most current T_2_ mapping techniques (6, 7) involve the acquisition of several thick 2D slices of the left-ventricular myocardium, which is largely adequate in the case of diseases that affect the entire myocardium or that have a well-defined pattern, such as acute ischemic myocardial injury. However, in a spectrum of inflammatory myocardial injuries such as viral myocarditis and sarcoidosis, the inflammation pattern is essentially irregular and unpredictable, despite typical patterns of segmental and transmural distribution. This may render the standard 2D T_2_ mapping technique suboptimal, since the disease foci can be missed due to insufficient coverage. Moreover, the thick slices may mask the disease foci through partial volume effect by including healthy and injured tissue in the same voxels. On the other hand, scanning would need to be prolonged to a large series of breath holds to cover the entire myocardium. To address these challenges, free-breathing high-resolution 3D T_2_ mapping techniques have been proposed in recent years (8–11), and have for example been applied in patients with graft rejection (12) and myocarditis (13).

Among these techniques, T_2_ mapping based on a self-navigated 3D radial acquisition (14, 15) can make use of the intrinsic robustness of 3D radial imaging against undersampling and motion artifacts, but it faces a challenge in its low effective signal-to-noise ratio (SNR), which leads to a loss in precision of the T_2_ maps. This 3D radial T_2_ mapping has therefore mostly been applied at a magnetic field strength of 3T (8, 12), and not at 1.5T, which may be more commonly used for cardiac magnetic resonance (CMR). However, recently several new techniques have been developed that can be used to increase the precision of a 3D radial T_2_ mapping, including resolving the motion instead of correcting it (16) in order to reduce motion streaking artifacts, and using compressed sensing (17) in order to reduce undersampling artifacts and to denoise the source images.

In this study, we therefore aimed to enable 3D radial T_2_ mapping at 1.5T by improving the image reconstruction, and to demonstrate the efficacy of this reconstruction method in healthy volunteers as well as in a small cohort of patients with suspected acute non-ischemic myocardial injury. To this end, the T_2_ maps were generated by first reconstructing respiratory motion-resolved source images, which were then registered to one another to decrease noise and motion artifacts, and thus to improve 3D T_2_ mapping precision and accuracy at 1.5T. These 3D T_2_ maps were then compared to routine 2D maps acquired in the same subjects.

## Materials and Methods

### Study Participants

This study was approved by the Institutional Review Board of the Medical University of Gdansk (#NKBBN/72/2019). All participants provided written informed consent prior to the procedure and none of them had contraindications for MRI.

To study the baseline relaxation times, healthy volunteers (*n* = 9, age = 43 ± 7 y, 5(56%) women, Table 1) without any history or symptoms of cardiovascular disease were recruited.

**Table 1:** Subject characteristics.

	**Healthy volunteers**	**Patients**
	**(*n* = 9)**	**(*n* = 25)**
Age, mean (SD) [years]	43(7)	49(11)
Gender, F *n*(%)	5 (56%)	9 (36%)
Heart rate, mean (SD) [bpm]	68 (11)	67 (13)
BMI, mean (SD) [kg/m^2^]	24.7 (1.0)	27.2 (4.2)
Hypertension, *n*(%)	0	15(60)
Diabetes, *n*(%)	0	3(12)
Hyperlipidemia, *n*(%)	0	6(24)
CAD, *n*(%)	0	3(12)
LV end-diastolic volume index, mean (SD) [ml/m^2^]	71(10)	85(30)
LV end-systolic volume index, mean (SD) [ml/m^2^]	26(7)	39(23)
LV ejection fraction, mean (SD) [%]	65(5)	55(9)
LV mass index, mean (SD) [g/m^2^]	59(6)	72(18)
Referral diagnosis, *n*(%)		
Sarcoidosis	NA	19(76)
Systemic sclerosis	NA	2(8)
Acute graft rejection	NA	2(8)
Myocarditis	NA	2(8)

*BMI, body mass index; CAD, coronary artery disease; LV, left ventricular; SD, standard deviation*.

Consecutive patients with suspected acute non-ischemic myocardial injury (*n* = 25, 19 cardiac sarcoidosis, 2 acute graft rejection, 2 systemic sclerosis, 2 myocarditis; 9 (36%) women, age 49 ± 10 y; Table 1) were recruited.

### MR Acquisition

All MR scanning was performed on a 1.5T clinical scanner (MAGNETOM Aera, Siemens Healthcare, Erlangen, Germany). All participants underwent routine bSSFP cine imaging to assess cardiac function (18), routine breath-held 2D T_2_ mapping (19), and the prototype free-breathing 3D T_2_ mapping. In addition, the patients underwent routine late gadolinium enhancement (LGE) imaging 7–15 min after injection of 0.1 mmol/kg of gadobutrol (Gadovist, Bayer AG, Leverkusen, Germany).

The routine T_2_ maps were acquired as ECG-triggered Cartesian 2D T_2_-prepared bSSFP images (6) with repetition time TR = 2.5 ms, echo time TE = 1.1 ms, flip angle = 70°, pixel bandwidth = 1,184 Hz/px, field of view = 360 × 288 mm^2^, slice thickness = 8 mm, acquired pixel size 2.49 × 1.88 mm^2^ interpolated to 1.88 × 1.88 mm^2^, T_2_ prep duration = 0/25/55 ms, breath-hold duration nine heartbeats (data acquired every three heartbeats), and GRAPPA acceleration factor 2. Images were acquired in a short-axis (SAX) orientation at the basal and mid-ventricular level. Since the reconstructed 2D maps were immediately available on the scanner, visibly corrupted maps were re-acquired as per routine protocol.

The free-breathing T_2_ maps were acquired as three ECG-triggered 3D radial bSSFP volumes with a phyllotaxis trajectory (14), TR = 2.6 ms, TE = 1.3 ms, flip angle = 35°, pixel bandwidth = 908 Hz/px, field of view = (220 mm)^3^, isotropic voxel size 1.6 mm^3^, T_2_ prep duration = 0/30/60 ms, and interleaves of 49 k-space lines acquired every other heartbeat preceded by a superior-inferior line that could be used for self-navigation (15). This resulted in a total acquisition time of 112 interleaves × 3 T_2_preps × 2 heartbeats/interleave = 672 heartbeats, or 11.2 min at 60 bpm.

Bloch equation simulations of the abovementioned proposed pulse sequence were performed for a heart rate range from 40 to 90 bpm with an assumed myocardial T_1_ relaxation of 1,050 ms and a true T_2_ of 50 ms to assess the influence of the heart rate on the estimated T_2_ relaxation time.

### T_2_ Map Reconstruction

The routine 2D T_2_ maps were reconstructed on the scanner (Siemens IDEA, Erlangen, Germany): the source images were non-rigidly registered (20) and a pixel-wise T_2_ fit was performed with the standard two-parameter exponential decay without offset, which resulted in maps in the DICOM format.

The respiratory motion-resolved reconstruction of 3D radial volumes (Supplementary Figure 1) was performed in MATLAB (the Mathworks, Natick, USA) on a workstation equipped with two Intel Xeon CPUs, 512 GB of RAM, and an NVIDIA Tesla K40 GPU. Here, a principal component analysis (PCA) was performed on the superior-inferior profiles in order to partition the dataset into four different respiratory states. 4D (x-y-z-respiratory dimensions) images were then reconstructed with a parallel imaging and compressed sensing algorithm that exploits sparsity along the respiratory dimension (16, 21, 22), resulting in separate images for all T_2_ preparation times and respiratory states. The compressed sensing optimization problem was solved with the conjugate gradient technique (17) using the finite difference operator as a sparsifying transform over the respiratory dimension with a weight λ. All respiratory bins were translationally and then non-rigidly registered to the end-expiration bin with Elastix (23), and were subsequently averaged in order to increase the SNR of each T_2_-prepared volume. After a second, similar, registration of the resulting three averaged T_2_-prepared volumes, voxel-wise T_2_ mapping with an offset factor to account for T_1_ recovery (24–26) was performed. Since the motion is no longer resolved after these registrations, we named the resulting 3D maps “motion-registered” T_2_ maps. The total reconstruction time from raw data to T_2_ map was recorded.

The regularization weight was optimized by comparing T_2_ map sharpness in maps reconstructed with λ = 0.01, λ = 0.05 (the commonly used value at this spatial resolution), and λ = 0.25 in a subgroup of *n* = 6 patients. T_2_ map sharpness was assessed by fitting a line from the middle of the septal myocardium to the left-ventricular blood pool with a parametrized sigmoid function (T_2_ (x) = a/(1+e^−*k*(*b*+*x*)^)+c), where a, b, and c are scaling variables and k is the sharpness (in px^−1^ or mm^−1^; higher is better) (27). The sharpness assessment was repeated for a total of five adjacent lines and the average sharpness k was reported. To ascertain that there is no significant difference between the proposed map reconstruction and alternatively first mapping the T_2_ relaxation time in each respiratory-resolved bin and then averaging these four bins, the T_2_ map sharpness was also quantified in this alternative reconstruction in these *n* = 6 patients.

In order to visually demonstrate that a self-navigated reconstruction (15) results in non-diagnostic maps at 1.5T due to too low SNR, a self-navigated reconstruction was performed in a single healthy volunteer, since the resulting maps were often too noisy for segmentation and quantitative analysis. This reconstruction was made with the same 3D radial data mentioned above. Here, the 1D displacement of the left-ventricular blood pool along the superior-inferior readouts acquired at the start of each interleave was used to correct each interleave for respiratory motion in k-space prior to image reconstruction (15, 28). The resulting three 3D images were translationally and then non-rigidly registered with Elastix, and voxel-wise T_2_ mapping (8) was performed.

### Map Analysis and Statistics

The visible myocardium in the routine 2D maps and their matching single slices in the 3D volumes were segmented in MATLAB. The T_2_ values of the entire visible myocardium and the regional segments defined by the American Heart Association (AHA) (29) were then measured in all volunteers and patients by two independent observers (JF and AS, with 7 and 10 years of experience with cardiac MRI, respectively). For the whole myocardium and 12 out of 16 AHA segments of each subject (2D apical segments were not included as they are generally considered prone to partial volume effects that may compromise the measurement accuracy) the T_2_ values, coefficients of variation (CoV, the standard deviation divided by the average), and the inter-subject standard deviation obtained with the two techniques were calculated. Since segmental values can strongly vary both due to difference in local disease patterns and precision of the technique, segmental T_2_ values were only directly compared in the healthy volunteers. In the patients, the segments that were LGE-positive were grouped for comparison with the LGE-negative segments. These values were then compared between the 2D and 3D techniques with paired Student's *t*-tests with a Bonferroni correction when appropriate, with *P* < 0.05 considered statistically significant when two quantities were compared. When multiple quantities were compared to one another a one-way ANOVA with a *post-hoc* Tukey correction for multiple comparisons was used.

The total number of segments that was not considered of diagnostic quality (i.e., not clearly defined or too thin for segmentation) by an experienced reader (KD) was counted for the 3D technique; this analysis could not be performed for the 2D technique, since visually corrupted maps were re-acquired, resulting in analyzable 2D T_2_ maps in all study subjects. The visual quality of the maps was assessed by two experienced CMR specialists (KD, AG) on a continuous scale from 1 to 10 with a visual analogue (30).

Bland-Altman analyses were performed to assess the T_2_ differences between the 2D and 3D techniques in the patients, as well as to assess the inter- and intra-observer agreement for the 3D technique. Trends in these Bland-Altman plots were tested for significance with Spearman's rank correlation.

## Results

**Figure 1:**
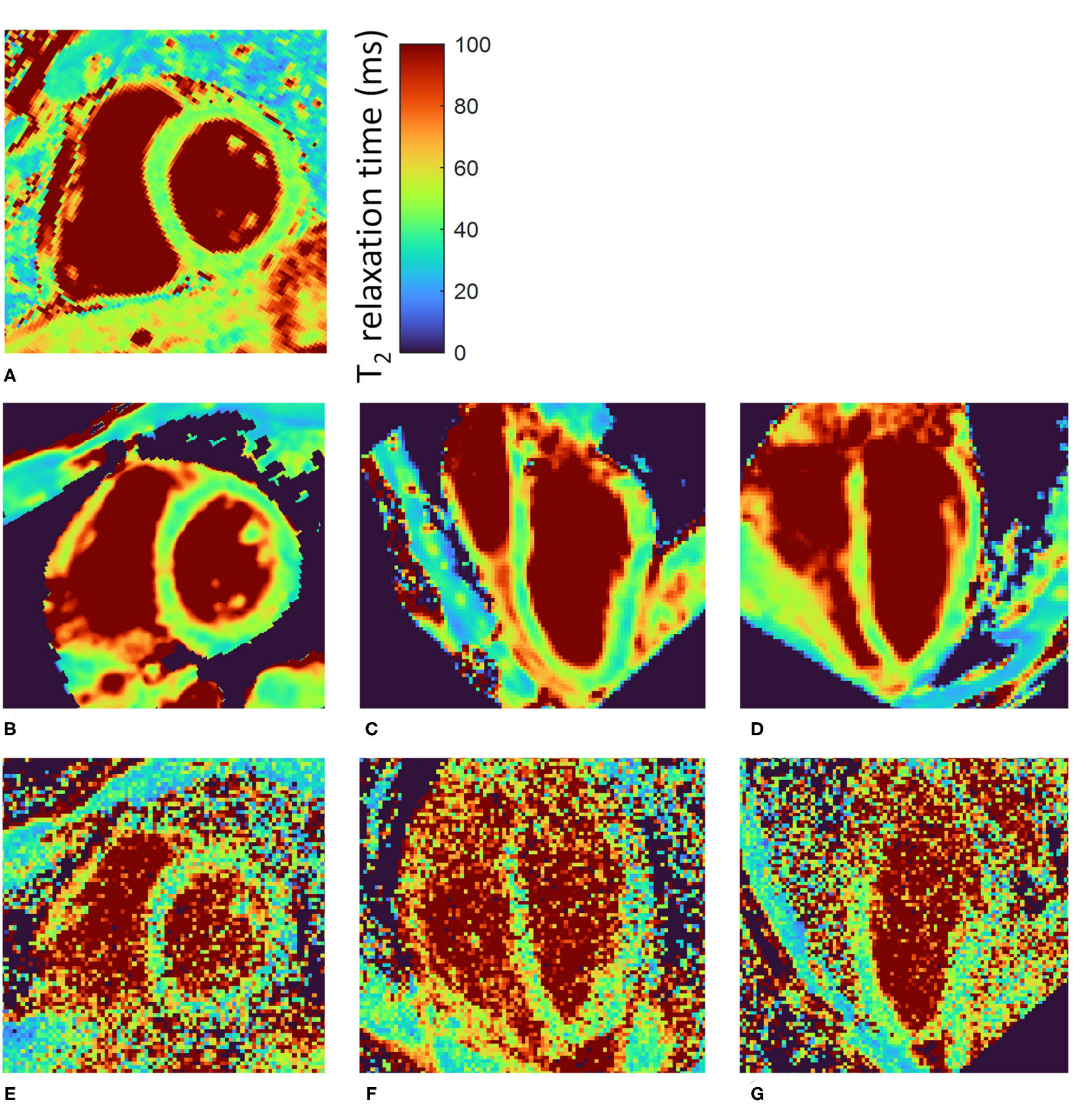
Motion-registered 3D T_2_ maps of the heart of a healthy volunteer. **(A)** Routine T_2_-prepared 2D bSSFP SAX T_2_ map. **(B)** Matching single slice from the motion-registered 3D T_2_ map; T_2_ values closely match those of the routine map. **(C,D)** Perpendicular long-axis (LAX) maps from the same 3D datasets as in **(B)**. **(E–G)** Three orthogonal slices from a self-navigated reconstruction at approximately the same locations as **(B–D)**. There is a slight orientation mismatch due to manual rotation of the volumes. The color bar shows the T_2_ relaxation time in ms.

The respiratory motion-resolved reconstruction resulted in visibly well-separated motion states in the source images (Supplementary Animated Figure 1), while motion-registered isotropic 3D T_2_ maps of the heart were successfully obtained in all subjects (Figure 1). Several features in these 3D maps were visually more blurred than their equivalents in the 2D maps. The self-navigated reconstruction of the source images did not lead to diagnostic maps (Figures 1E–G). The total reconstruction time from raw data to T_2_ map was 1 h 19 ± 2min.

The sharpness measurements in the patient subgroup resulted in k = 1.56±0.22 mm^−1^ for the proposed technique, i.e., respiratory-registered T_2_ maps with λ = 0.05 (Supplementary Figure 2). The motion-registered mapping with λ = 0.01 and λ = 0.25 resulted in k = 1.67 ± 0.34 mm^−1^ (*P* = 0.32) and k = 1.49 ± 0.29 mm^−1^ (*P* = 0.34), respectively. Mapping each respiratory-resolved bin first and then averaging these maps as an alternative reconstruction resulted in k = 1.63 ± 0.29 mm^−1^ (*P* = 0.40) for the motion-registered T_2_ mapping. Conversely, the entire patient group resulted in k = 1.71 ± 0.29 mm^−1^ and k = 1.89 ± 0.40 mm^−1^ (*P* = 0.038) for the 3D and 2D techniques respectively, demonstrating the higher sharpness in the 2D technique despite the larger pixel size. This also held true in the healthy volunteers at k = 1.71 ± 0.31 vs. k = 1.98 ± 0.38 mm^−1^ (*P* = 0.037).

**Figure 2:**
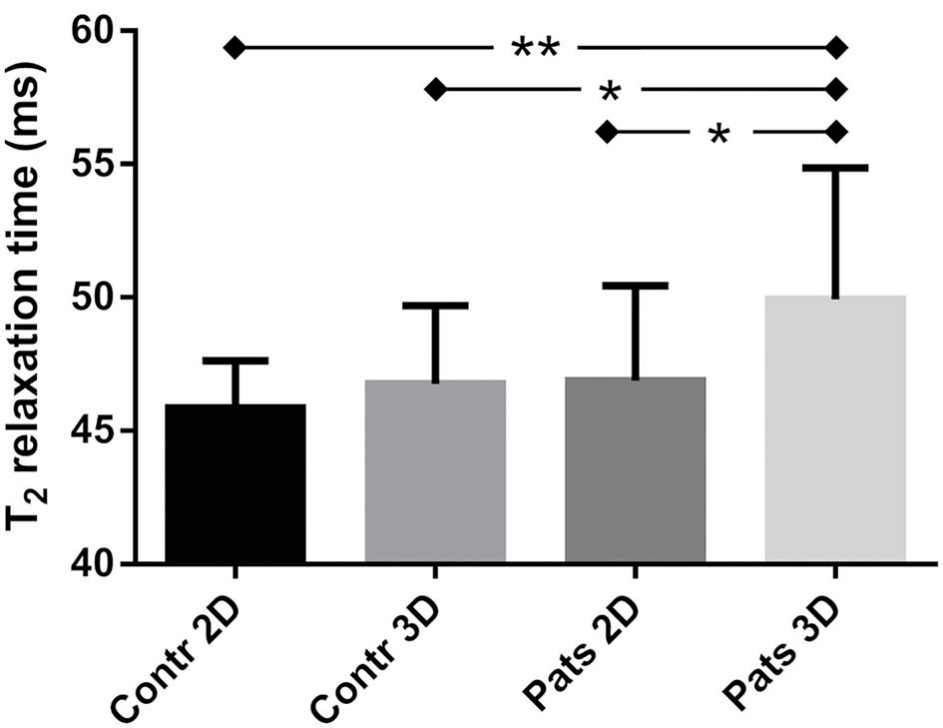
Myocardial T_2_ relaxation times obtained with the 2D and 3D T_2_ mapping techniques in the left ventricle of healthy controls (Contr) and patients (Pats). There was a small but significant difference between the two groups as quantified with the 3D technique, but not with the 2D technique. Within the patient group, the 3D technique also resulted in a small difference compared to the 2D technique. *indicates *p* < 0.05, **indicates *p* < 0.01.

**Figure 3:**
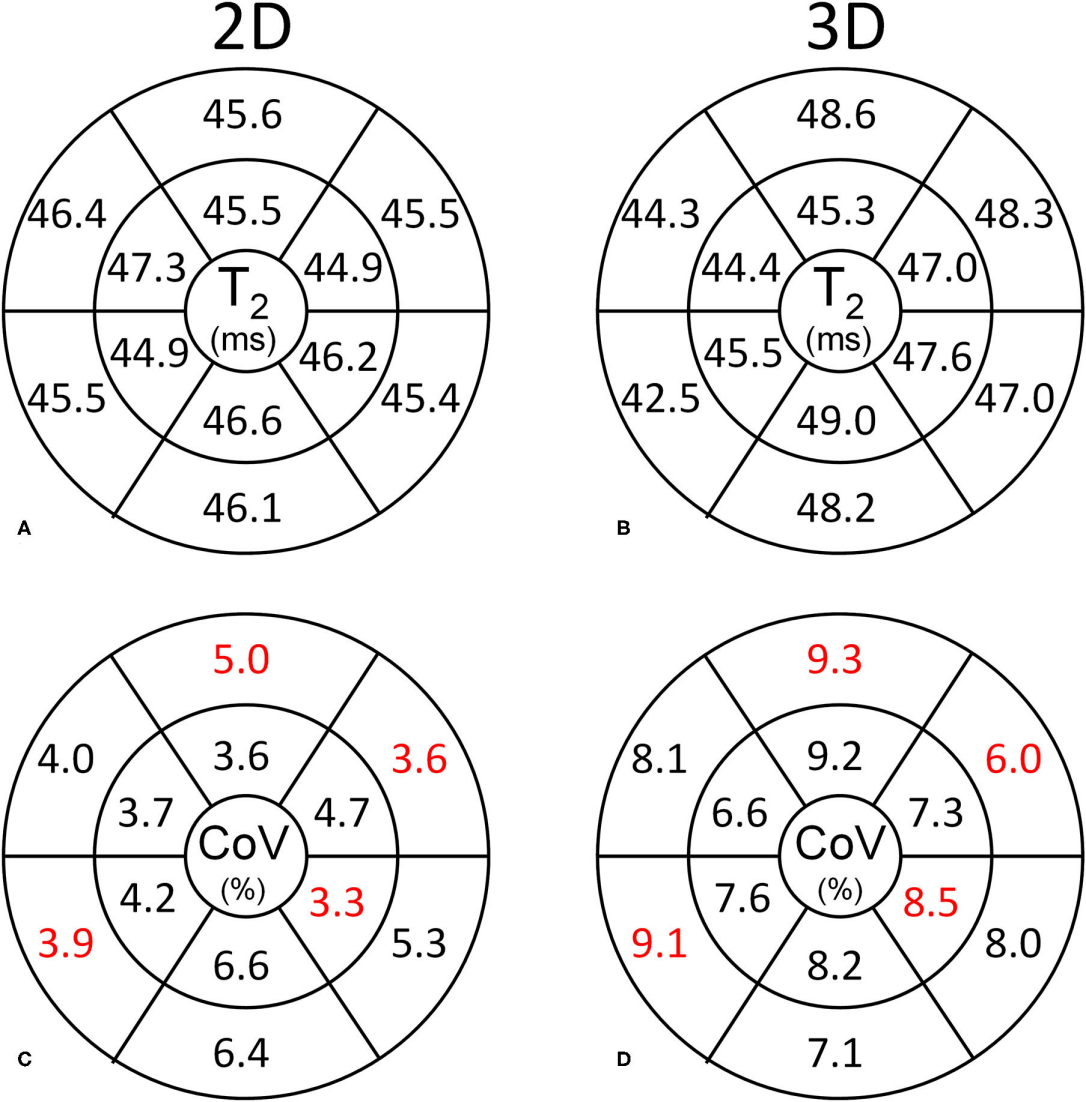
Segmental analysis of the myocardial T_2_ values in the healthy volunteers. Basal and mid-ventricular segmental T_2_ values and CoVs are shown according to the standard AHA segmentation. **(A,B)** The segmental T_2_ values obtained with the 2D and 3D techniques are highly similar. **(C,D)** The CoV is consistently lower for the 2D technique than for the 3D technique. Significantly different CoV values between the respective segments are marked in red.

In the healthy volunteers, the myocardial T_2_ values obtained with the 2D and 3D techniques were highly similar at 45.8 ± 1.8 and 46.8 ± 2.9 ms (*P* = 0.33, Figure 2), respectively, while the CoV was lower in the 2D technique at 4.5 ± 0.8 vs. 8.2 ± 1.5% for the 3D technique (*P* ≤ 0.001). The segmental T_2_ values did not significantly differ between the two techniques (*P* ≥ 0.09 for all, Figures 3A,B), while the CoV differed in 4 out of 12 segments (Figures 3C,D). Out of 108 analyzed segments in the 3D T_2_ maps in healthy volunteers, 6 (5.6%, of which 4 [3.7%] in one subject) were deemed non-diagnostic due to inaccurately registered thin myocardium.

**Figure 4:**
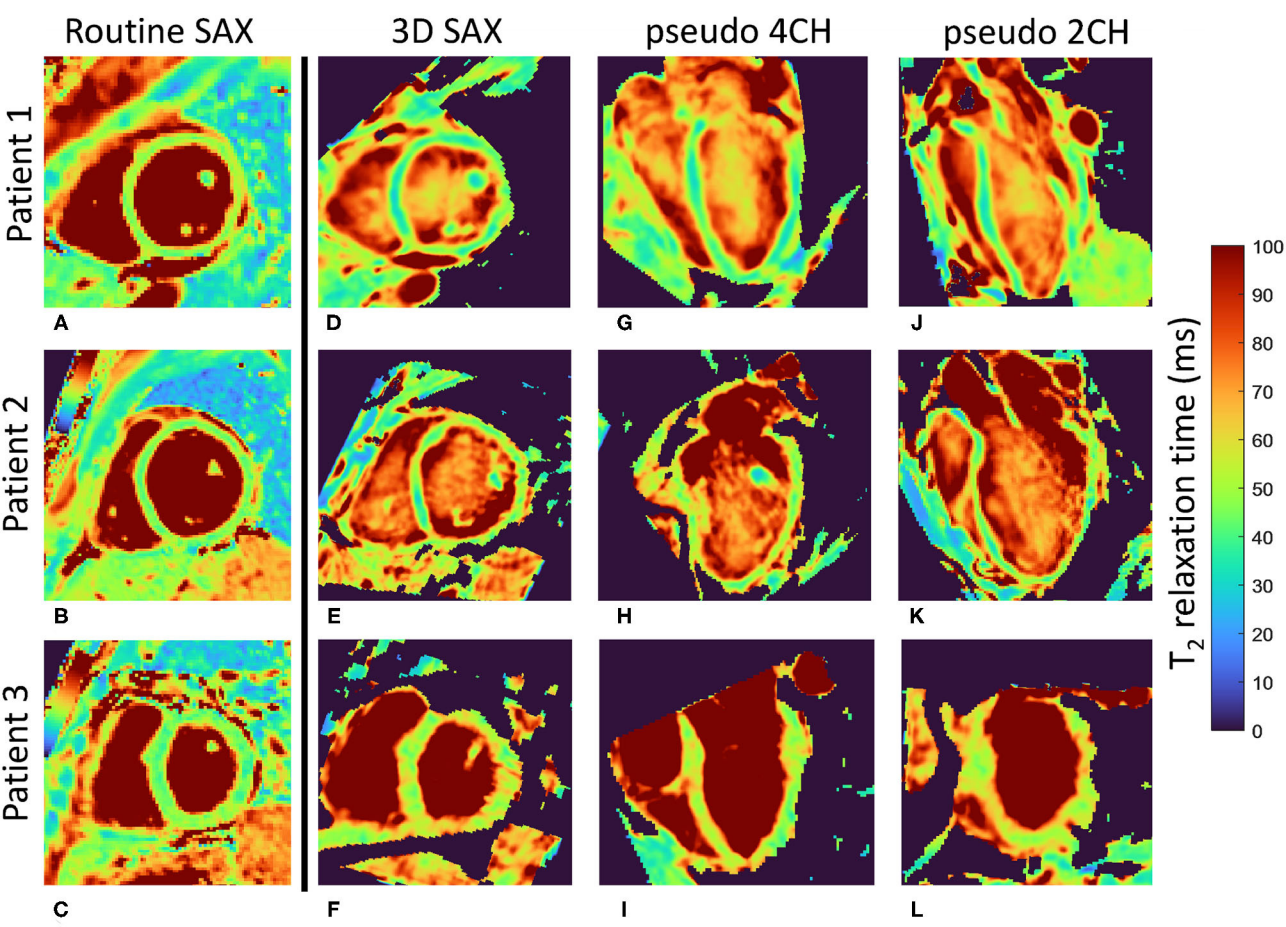
Motion-registered 3D T_2_ maps of the heart of three patients. **(A–C)** Routine T_2_-prepared 2D bSSFP short-axis (SAX) T_2_ maps. **(D–F)** Matching single slices from the motion-registered 3D T_2_ maps; T_2_ values closely match those of the routine maps. **(G–L)** Perpendicular long-axis (LAX) maps from the same 3D datasets as in **(D–F)**. Patient 1 (female, 37 y.o.) and patient 2 (male, 44 y.o.) had myocarditis, while patient 3 (male, 65 y.o.) had sarcoidosis. Local minor T_2_ fluctuation can be observed in all three orientations of the 3D T_2_ maps and all three patients, and might be caused by small misregistrations, as well as by local T_2_ variations that show up in the 1.6 mm-thick 3D slice, but are averaged out in the 8 mm-thick 2D slice. However, global T_2_ values did not significantly differ in any of the patients. The color bar shows the T_2_ relaxation time in ms.

**Figure 5:**
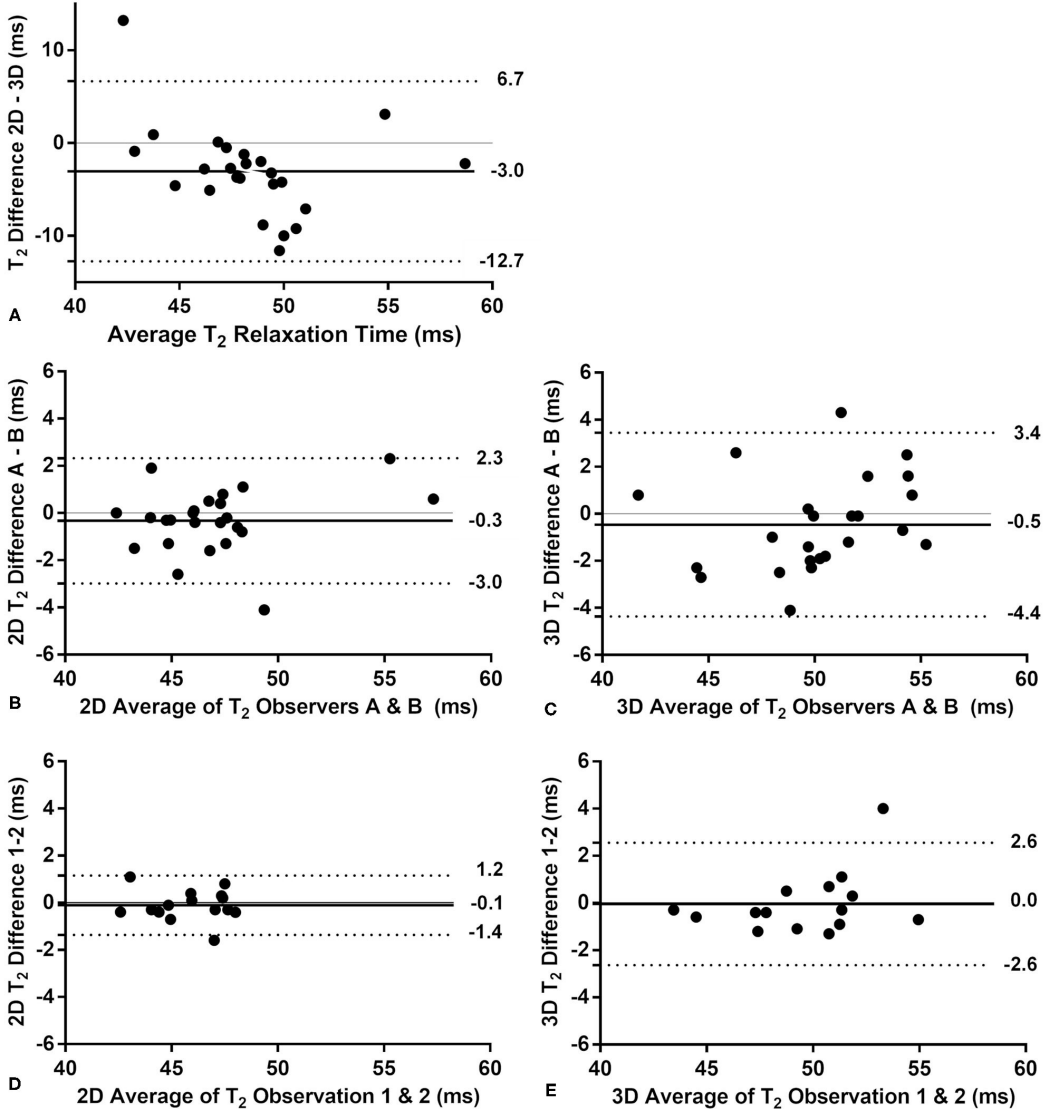
Bland-Altman analyses of the 2D vs. the 3D T_2_ mapping technique in the patients. The bias is indicated with a solid line, while the upper and lower limits of the 95% confidence interval are indicated with a dotted line. **(A)** The comparison of the 2D and 3D techniques shows a small bias, but no significant trend (*P* = 0.11 for a linear correlation). **(B)** The interobserver comparison for the routine 2D technique indicates a very small bias and small confidence interval. **(C)** The interobserver comparison of the 3D technique has a similarly small bias and a slightly larger confidence interval than the 2D technique. Neither interobserver plot shows a significant trend (*P* > 0.21). The intra-observer analysis performed in a subset of patients (*n* = 15) showed no bias or trend for the 2D **(D)** and 3D **(E)** techniques, although the confidence interval for the 3D technique was twice as large as that of the 2D technique.

In the patients, the average myocardial T_2_ relaxation times did differ between the 2D (46.7 ± 3.6 ms) and 3D techniques (50.1 ± 4.2 ms, *P* = 0.004, Figures 2, 4). The CoV was again lower in the 2D technique than in the 3D technique at 6.8 ± 1.5 vs. 10.4 ± 1.8% (*P* < 0.001). When the myocardium was segmented according to the AHA guidelines, the two techniques resulted in significantly different T_2_ values in the basal-anterior, basal-inferior, mid-inferoseptal and mid-inferior segments (segment numbers 1, 4, 9, and 10; *P* ≤ 0.001). Conversely, the segmental CoV in the patients was significantly lower for the 2D technique in all segments (*P* ≤ 0.001) except in the mid-anteroseptal segment. Out of the 300 myocardial segments analyzed in the patients, 14 (4.6%, of which 8 (2.6%) in one patient) were deemed non-diagnostic. A Bland-Altman analysis of the 2D vs. the 3D technique demonstrated that there was a small bias of −3.0 ms (Figure 5A). Although a slight trend can be observed for the difference to become more negative as the average increases, this trend was not significant (ρ = −0.33, *P* = 0.11). The patient T_2_ values as measured with the 2D technique were not significantly different from those in healthy volunteers (*P* = 0.50), while the difference was significant when measured with the 3D technique (*P* = 0.04).

Inter-observer analyses of the 2D and 3D techniques showed very small biases between the observers, no visible or significant trends (ρ ≤ 0.27, *P* ≥ 0.20), and similar confidence intervals (Figures 5B,C). The intra-observer analyses of a subset of patients (*n* = 15) showed a higher confidence interval for the 3D technique (Figures 5D,E) and no significant trend for either technique (ρ < 0.35, *P* > 0.2). The visual quality score was higher for the 2D maps than for the 3D maps at 9.6 ± 0.4 vs. 7.2 ± 2.1 (*P* < 0.001, Supplementary Figure 3).

**Figure 6:**
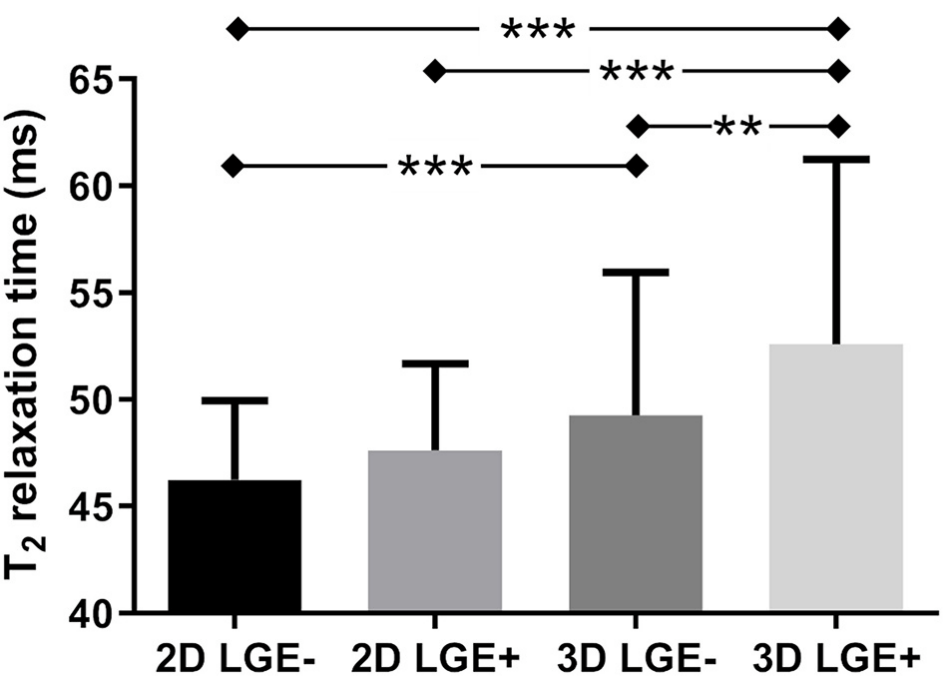
T_2_ relaxation times in myocardial segments with and without late gadolinium enhancement (LGE). The myocardium of the patients in the LGE images and T_2_ maps was segmented according to the AHA guidelines, and all segments of all patients that were LGE-positive (LGE+) were grouped, as were all LGE-negative (LGE–) segments. While there was no difference between the LGE– and LGE+ segments as quantified with the routine 2D technique, a small but significant difference was detected with the proposed 3D T_2_ mapping technique. **indicates *p* < 0.01, ***indicates *p* < 0.001.

**Figure 7:**
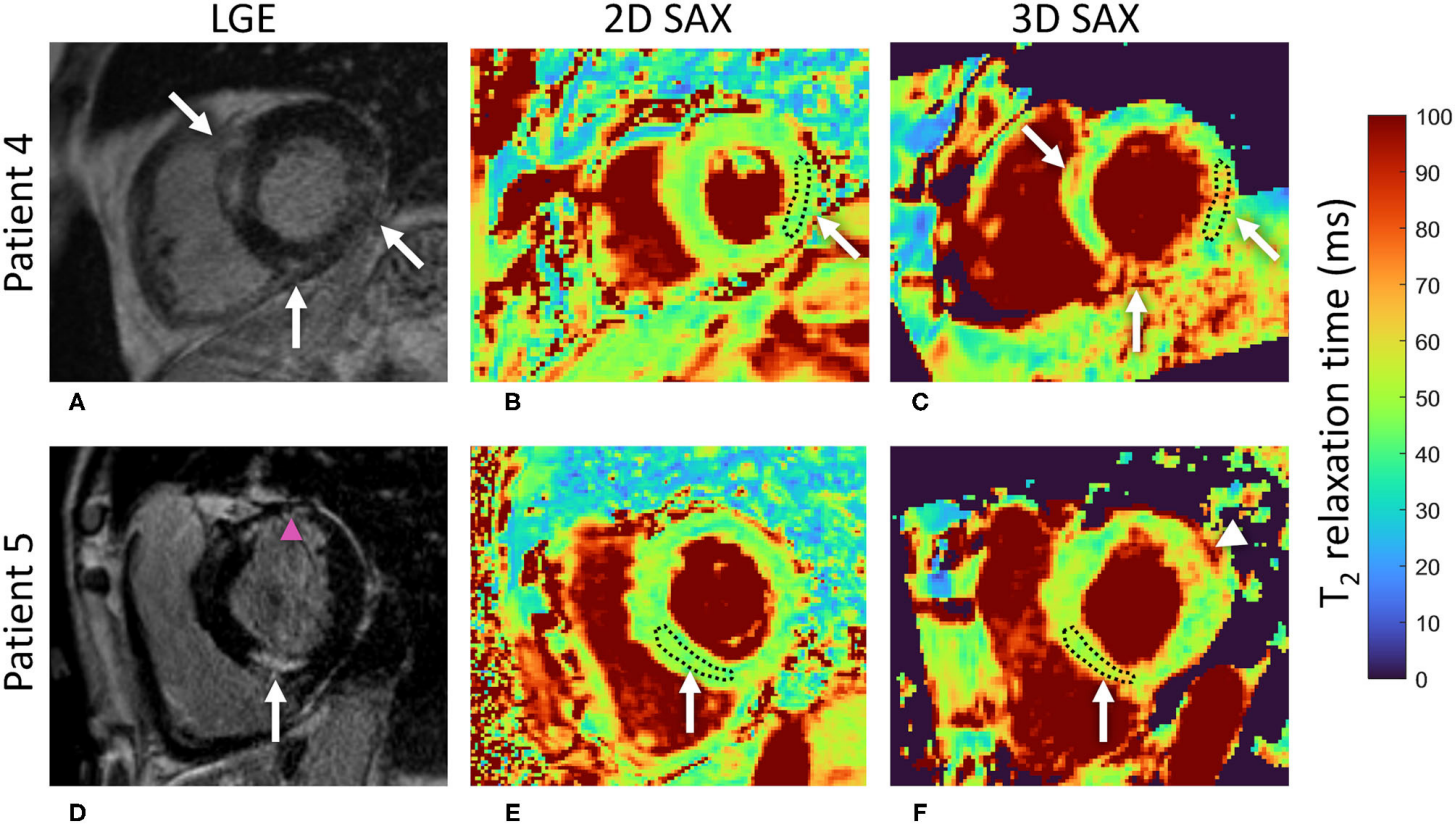
A comparison of the 2D and 3D T_2_ mapping techniques in slices with lesions detected with LGE imaging. **(A)** LGE shows intramyocardial areas of irreversible damage in several basal regions of the LV in a 56 y.o. male patient with suspected cardiac sarcoidosis. **(B)** No T_2_ elevation is visually apparent on the 2D T_2_ map. As an example, the measured segmental ROI average T_2_ in the basal inferolateral segment was 46 ms. **(C)** Conversely, local increases of the T_2_ relaxation time can be spotted on the 3D T_2_ map in areas corresponding to the LGE regions (white arrows). The measured segmental ROI average T_2_ in the respective inferolateral segment was 56 ms, suggestive of an area of acute injury or ongoing inflammation. **(D)** An intramyocardial area of irreversible damage as shown by LGE (left, white arrow) in the basal inferoseptal segment of the LV in a 66 y.o. male patient with suspected cardiac sarcoidosis. The pink arrowhead indicates an ischemic scar in the anterior wall. **(E)** Again, no T_2_ elevation was visually apparent on the 2D T_2_ map in the respective segment with the segmental ROI average T_2_ = 47 ms. **(F)** Local (white arrow) increase of the T_2_ relaxation time (segmental ROI average T_2_ = 55 ms) can be spotted in this region on the 3D T_2_ map, suggestive of an area of acute injury. White arrowhead points to another area of elevated T_2_ that does not correspond to any LGE in **(D)**. The institutional reference range for 2D T_2_ mapping is 44 (2.4) ms, i.e., 39–49 ms. LGE, late gadolinium enhancement; LV, left ventricle; ROI, region of interest.

The LGE-positive segments did not have a significantly higher T_2_ relaxation when compared to their LGE-negative counterparts as quantified with the 2D technique (T_2LGE−_ = 46.2 ± 3.7 vs. T_2LGE+_ = 47.6 ± 4.1 ms, *P* = 0.49, Figures 6, 7). Conversely, the 3D technique did result in a significant difference (T_2LGE−_ = 49.3 ± 6.7 vs. T_2LGE+_ = 52.6 ± 8.7 ms, *P* = 0.006) despite its larger spread in individual T_2_ values. Both 2D segment groupings were also significantly different from their 3D equivalents (*P* < 0.001). The Bloch equation simulations indicated that the proposed 3D mapping technique will moderately underestimate (<5%) the T_2_ relaxation times for high heart rates (80–90 bpm, Supplementary Figure 4).

## Discussion

In this study, we proposed a novel method to obtain high-resolution respiratory motion-corrected 3D T_2_ maps of the heart at 1.5T by extracting, resolving, and then registering the respiratory motion states, and tested this technique in patients and healthy volunteers. The respiratory motion was detected and visually resolved well in all subjects, and the resulting isotropic 3D T_2_ values matched their routine 2D counterparts while also enabling the visualization of other views of the heart.

The T_2_ CoV (i.e., the inverse of precision) agreed well with that obtained in previous 3D T_2_ mapping studies (8, 12), but was higher than commonly reported for 2D T_2_ mapping (31, 32). The main two causes of this are most likely the (1.9 × 1.9 × 8)/1.6^3^ = 7 times smaller voxel size and the radial acquisition itself, although this is partially compensated by the denoising effect of the compressed-sensing reconstruction and the 3D nature of the acquisition. The higher CoV values that are seen in some segments might result from these particular segments being more prone to small misregistrations due to the neighboring epicardial fat or small myocardial thickness. If a higher precision is desired, it can most likely be achieved by increasing the voxel size, the acquisition time, or the regularization parameters of the reconstruction. Similarly, the observed residual blurring in the short-axis plane could for example be caused by incorrect motion extraction or misregistration. The slightly (but not significantly) different T_2_ averages in the healthy volunteers with the 2D and 3D techniques most likely also have several origins, such as a different interplay with the T_1_ relaxation time during the acquisition (24), and a residual noise floor that promotes increased T_2_ relaxation time measurements (25). The observed map sharpness agrees well with previous map sharpness quantifications (33), while the minor (and non-significant) differences between the reconstructions with different regularization weights suggest a relatively broad optimum for the regularization parameter. The alternative reconstruction where T_2_ maps were obtained for the four respiratory motion bins, followed by averaging of these bins, also led to similar map sharpness, though at the cost of 4-fold longer map fitting time.

It is well-known that T_2_ reference values in healthy subjects may vary and that they are therefore not easily comparable between studies (34–36). In addition to magnetic field strength, pulse sequence type, and specific parameters (32), other factors play a role, including gender and cardiac physiology (34). Therefore, the assessment of institution-specific reference ranges with established scanner- and sequence-specific T_2_ values in healthy volunteers are strongly recommended (2). With scanner- and sequence-specific reference values as a prerequisite, it was demonstrated that T_2_ values in studies of specific inflammatory conditions such as myocarditis (3), systemic sclerosis (37), and sarcoidosis (35) can unequivocally be used to identify myocardial injury. Therefore, the significant difference in T_2_ relaxation time that was found in patients using the 3D but not the 2D T_2_ mapping merits special attention, as this might suggest better sensitivity to detect tissue alterations with the proposed high-resolution motion-registered radial 3D T_2_ mapping technique at 1.5T. However, confounders such as the observed blurring, SNR, irregular breathing patterns of the patients, and the heart-rate dependence of the pulse sequence should also be taken into consideration. Of note, the patient population in our study was a fairly “healthy” patient population that included consecutive patients with suspected myocardial inflammation, where no large elevation of T_2_ was observed by conventional protocol. Therefore, the significantly higher T_2_ values in 3D maps in patients that were not seen in conventional 2D maps and were higher than 3D T_2_ values in controls suggest potential for the detection of more subtle changes or earlier stages of disease. Further study will be required in a broader spectrum of disease severity to shed more light on these findings. The proposed 3D technique indicated a small but significant T_2_ difference between the LGE+ and LGE– myocardial segments, while the routine 2D technique did not. This may again have a technical origin such as the smaller voxels in the 3D maps having less partial volume effect (especially through the much thinner slices) with healthy tissue that blunts the signal change and therefore reduces the sensitivity. Even though LGE presence by itself does not directly indicate edema or inflammation, it potentially colocalizes with patches of active disease in patients with an ongoing disease process. Thus, the slightly increased T_2_ value in LGE+ segments suggests that smaller and isotropic voxels may be more appropriate in patients with potentially focal non-ischemic injury. Nevertheless, a higher sensitivity of 3D T_2_ mapping would need to be confirmed with a clinical standard method such as positron emission tomography (38). While we did not evaluate short-axis apical segments in this study, it may be of interest to include these in future studies to take advantage of a reduced partial volume effect consistent with smaller voxel sizes.

The acquisition itself could be improved in several ways in future studies. By using a different radial k-space trajectory for each T_2_-prepared volume (39), the sparsity in the relaxation time dimension could also be exploited for a compressed sensing reconstruction (11), which should result in a higher precision. Calculating the T_2_ in each pixel based on an individually patient-specific simulated dictionary (40, 41) could also be used to remove the constraint of acquiring every other heartbeat, which would significantly accelerate the acquisition, and would remove the heart-rate dependence of the T_2_ relaxation time.

The higher variation in quality of the 3D maps as compared to the 2D maps is not surprising, as the 2D T_2_ maps were immediately re-acquired during the scanning sessions if they were visually of unsatisfactory quality, which was not feasible for 3D T_2_ maps because of time constraints. Of note, most of the segments that were termed non-diagnostic belonged to a single patient and a single volunteer, which suggests that there might have been a subject-specific challenge involved, such as particularly thin myocardium or a sub-optimal ECG triggering. Therefore, considering the inherent complexity of the isotropic 3D sequence and multiple reconstruction steps required in the proposed technique, the observed quality score scatter is an expected trade-off, especially with the improvement potential outlined above. Studies with a combination of pseudo-spiral Cartesian trajectory (instead of the 3D radial used here), a 2D respiratory motion correction (instead of the 1D correction), and the addition of patch-based denoising (which improves the apparent SNR) did result in consistent T_2_ precision (11, 13). The patch-based denoising (42) in particular could improve the 3D T_2_ mapping presented here, since it might improve the apparent SNR of the source images, resulting in better image registration as well as improved map precision.

In conclusion, respiratory motion-registered 3D radial imaging at 1.5T led to accurate isotropic 3D whole-heart T_2_ maps, both in the healthy volunteers and in a small patient cohort with suspected inflammatory myocardial injury. With the 3D technique, significantly higher T_2_ values were found in patients as compared to controls, as well as in LGE-positive as compared to LGE-negative segments, both of which were not observed with the routine 2D technique. These findings are suggestive of the technique's potential to increase the sensitivity of CMR for localized inflammatory myocardial injury. Further study will be needed in a broader spectrum of disease severity to demonstrate its clinical utility.

## Data Availability Statement

The raw data supporting the conclusions of this article will be made available by the authors, without undue reservation.

## Ethics Statement

The studies involving human participants were reviewed and approved by Institutional Review Board of the Medical University of Gdansk. The patients/participants provided their written informed consent to participate in this study.

## Author Contributions

KD and RBvH designed the study. LD, CR, DP, MS, JY, and RBvH developed and implemented the pulse sequence and image reconstruction algorithm. KD, JF, and HJ recruited the volunteers and patients and performed their diagnoses. AS and AG performed the MR scanning. RBvH supervised the analysis of MRI results and the manuscript draft. KD, KG, AS, ES, and RBvH analyzed the results and performed the statistical analysis. All authors participated in manuscript drafting and editing, approved the final version of the submitted manuscript, and agreed to the submission of the manuscript to Frontiers in Cardiovascular Medicine.

## Funding

This study was funded by grants from the Swiss Heart Foundation and the Swiss National Science Foundation (grant number 32003B_182615) to RH, as well as statutory grant (ST-98) of the Medical University of Gdansk, Poland to KD and HJ.

## Conflict of Interest

DP and KG are full-time employees of Siemens Healthcare. The remaining authors declare that the research was conducted in the absence of any commercial or financial relationships that could be construed as a potential conflict of interest.

## Publisher's Note

All claims expressed in this article are solely those of the authors and do not necessarily represent those of their affiliated organizations, or those of the publisher, the editors and the reviewers. Any product that may be evaluated in this article, or claim that may be made by its manufacturer, is not guaranteed or endorsed by the publisher.

## Supplementary Material

Supplementary Figure 1 A flowchart of the proposed 3D T_2_ mapping method. Data are indicated in colored boxes, while manipulations are mentioned as unboxed text.

Supplementary Figure 2 Sharpness measurement and various reconstructions of a 3D T_2_ map. **(A)** A T_2_ map in grayscale with a display range from 0 to 300 ms to better visualize the blood pool, with red crosses indicating the start- and end-points of the assessed transition. Four horizontal pixel lines below it were also assessed. **(B)** Normalized T_2_ values in the pixel (blue circles) and the parametrized sigmoid fit (red curve). The sharpness of the transition was characterized by k = 2.6 px^−1^, which at the 1.6 mm spatial resolution translated to k = 1.6 mm^−1^. This value was averaged with those obtained from the lines below it. **(C)** The proposed 3D T_2_ mapping method in another patient. **(D)** An alternative reconstruction method in which the individual respiratory bins are all mapped first, and these bin maps are then averaged. While the papillary muscle appears to resolve better, the sharpness of the blood-myocardium interface remains constant. **(E,F)** The proposed reconstruction method, but with λ = 0.01 and 0.25. The blood-myocardium interfaces appear marginally sharper and more blurred, respectively, but this is balanced by respectively increased and decreased T_2_ variation in the myocardium.

Supplementary Figure 3 A paired comparison of the visual quality scores as agreed by two experienced readers. The 3D T_2_ maps have a much higher variation in quality than the 2D T_2_ maps (7.2 ± 2.1 vs. 9.6 ± 0.4, *P* < 0.001), although it should be noted that the 2D T_2_ maps were immediately re-acquired during the scanning sessions if they were visually of unsatisfactory quality.

Supplementary Figure 4 A Bloch equation simulation of the influence of the patient's heart rate on the estimated T_2_ value. Since sampling occurs every other heartbeat, T_1_ relaxation will cause a gradual underestimation of the T_2_ value with the increase of the heart rate.

Supplementary Animated Figure 1 An animation of the respiratory-resolved reconstruction of a source image with T_2_prep duration of 30 ms in a patient. The breathing motion is well-resolved, and results in a non-rigid translation of the heart.

## References

[1] Friedrich MG. Myocardial edema–a new clinical entity? Nat Rev Cardiol. (2010) 7:292–6. 10.1038/nrcardio.2010.28 20309007

[2] Messroghli DR Moon JC Ferreira VM Grosse-Wortmann L He T Kellman P. Clinical recommendations for cardiovascular magnetic resonance mapping of T_1_, T_2_, T_2_^*^ and extracellular volume: a consensus statement by the Society for Cardiovascular Magnetic Resonance (SCMR) endorsed by the European Association for Cardiovascular Imaging (EACVI). J Cardiovasc Magn Reson. (2017) 19:75. 10.1186/s12968-017-0389-8 28992817 PMC5633041

[3] Lurz P Luecke C Eitel I Föhrenbach F Frank C Grothoff M. Comprehensive cardiac magnetic resonance imaging in patients with suspected myocarditis. J Am Coll Cardiol. (2016) 67:1800–11. 10.1016/j.jacc.2016.02.013 27081020

[4] Markousis-Mavrogenis G Bournia V-K Panopoulos S Koutsogeorgopoulou L Kanoupakis G Apostolou D. Cardiovascular magnetic resonance identifies high-risk systemic sclerosis patients with normal echocardiograms and provides incremental prognostic value. Diagnostics. (2019) 9:220. 10.3390/diagnostics9040220 31835765 PMC6963862

[5] Puntmann VO Isted A Hinojar R Foote L Carr-White G Nagel E. T1 and T2 mapping in recognition of early cardiac involvement in systemic sarcoidosis. Radiology. (2017) 285:63–72. 10.1148/radiol.2017162732 28448233

[6] Giri S Chung YC Merchant A Mihai G Rajagopalan S Raman SV. T2 quantification for improved detection of myocardial edema. J Cardiovasc Magn Reson. (2009) 11:56. 10.1186/1532-429X-11-56 20042111 PMC2809052

[7] Sprinkart AM Luetkens JA Träber F Doerner J Gieseke J Schnackenburg B. Gradient Spin Echo (GraSE) imaging for fast myocardial T2 mapping. J Cardiovasc Magn Reson. (2015) 17:12. 10.1186/s12968-015-0127-z 25885268 PMC4326516

[8] van Heeswijk RB Piccini D Feliciano H Hullin R Schwitter J Stuber M. Self-navigated isotropic three-dimensional cardiac T2 mapping. Magn Reson Med. (2015) 73:1549–54. 10.1002/mrm.25258 24809849

[9] Ding H Fernandez-de-Manuel L Schar M Schuleri KH Halperin H He L. Three-dimensional whole-heart T2 mapping at 3T. Magn Reson Med. (2015) 74:803–16. 10.1002/mrm.25458 25242141

[10] Yang HJ Sharif B Pang J Kali A Bi X Cokic I. Free-breathing, motion-corrected, highly efficient whole heart T mapping at 3T with hybrid radial-cartesian trajectory. Magn Reson Med. (2015) 75:126–36. 10.1002/mrm.25576 25753385 PMC4561222

[11] Bustin A Milotta G Ismail TF Neji R Botnar RM Prieto C. Accelerated free-breathing whole-heart 3D T _2_ mapping with high isotropic resolution. Magn Reson Med. (2020) 83:988–1002. 10.1002/mrm.27989 31535729 PMC6899588

[12] van Heeswijk RB Piccini D Tozzi P Rotman S Meyer P Schwitter J. Three-dimensional self-navigated T2 mapping for the detection of acute cellular rejection after orthotopic heart transplantation. Transplant Direct. (2017) 3:e149. 10.1097/TXD.0000000000000635 28405605 PMC5381742

[13] Bustin A Hua A Milotta G Jaubert O Hajhosseiny R Ismail TF. High-spatial-resolution 3D whole-heart MRI T2 mapping for assessment of myocarditis. Radiology. (2021) 298:578–86. 10.1148/radiol.2021201630 33464179 PMC7924517

[14] Piccini D Littmann A Nielles-Vallespin S Zenge MO. Spiral phyllotaxis: the natural way to construct a 3D radial trajectory in MRI. Magn Reson Med. (2011) 66:1049–56. 10.1002/mrm.22898 21469185

[15] Piccini D Littmann A Nielles-Vallespin S Zenge MO. Respiratory self-navigation for whole-heart bright-blood coronary MRI: methods for robust isolation and automatic segmentation of the blood pool. Magn Reson Med. (2012) 68:571–9. 10.1002/mrm.23247 22213169

[16] Di Sopra L Piccini D Coppo S Stuber M Yerly J. An automated approach to fully self-gated free-running cardiac and respiratory motion-resolved 5D whole-heart MRI. Magn Reson Med. (2019) 82:2118–32. 10.1002/mrm.27898 31321816

[17] Lustig M Donoho D Pauly JM. Sparse MRI: the application of compressed sensing for rapid MR imaging. Magn Reson Med. (2007) 58:1182–95. 10.1002/mrm.21391 17969013

[18] Kramer CM Barkhausen J Flamm SD Kim RJ Nagel E Society for Cardiovascular Magnetic Resonance Board of Trustees Task Force on Standardized Protocols. Standardized cardiovascular magnetic resonance (CMR) protocols 2013 update. J Cardiovasc Magn Reson. (2013) 15:91. 10.1186/1532-429X-15-91 24103764 PMC3851953

[19] Huang TY Liu YJ Stemmer A Poncelet BP. T2 measurement of the human myocardium using a T2-prepared transient-state TrueFISP sequence. Magn Reson Med. (2007) 57:960–6. 10.1002/mrm.21208 17457877

[20] Chefd'hotel C Hermosillo G Faugeras O. Flows of diffeomorphisms for multimodal image registration. In: Proceedings IEEE International Symposium on Biomedical Imaging. Washington, DC (2002). p. 753–6.

[21] Piccini D Feng L Bonanno G Coppo S Yerly J Lim RP. Four-dimensional respiratory motion-resolved whole heart coronary MR angiography. Magn Reson Med. (2017) 77:1473–84. 10.1002/mrm.26221 27052418 PMC5040623

[22] Feng L Coppo S Piccini D Yerly J Lim RP Masci PG. 5D whole-heart sparse MRI. Magn Reson Med. (2018) 79:826–38. 10.1002/mrm.26745 28497486 PMC5681898

[23] Klein S Staring M Murphy K Viergever MA Pluim JPW. elastix: a toolbox for intensity-based medical image registration. IEEE Trans Med Imaging. (2010) 29:196–205. 10.1109/TMI.2009.2035616 19923044

[24] van Heeswijk RB Feliciano H Bongard C Bonanno G Coppo S Lauriers N. Free-breathing 3 T magnetic resonance T2-mapping of the heart. JACC Cardiovasc Imaging. (2012) 5:1231–9. 10.1016/j.jcmg.2012.06.010 23236973

[25] Bano W Feliciano H Coristine AJ Stuber M van Heeswijk RB. On the accuracy and precision of cardiac magnetic resonance T2 mapping: A high-resolution radial study using adiabatic T2 preparation at 3 T. Magn Reson Med. (2017) 77:159–69. 10.1002/mrm.26107 26762815

[26] Colotti R Omoumi P Bonanno G Ledoux J-B van Heeswijk RB. Isotropic three-dimensional T2 mapping of knee cartilage: Development and validation. J Magn Reson Imaging. (2018) 47:362–71. 10.1002/jmri.25755 28489309

[27] Ahmad R Ding Y Simonetti OP. Edge sharpness assessment by parametric modeling: Application to magnetic resonance imaging: EDGE SHARPNESS ASSESSMENT FOR MRI. Concepts Magn Reson. (2015) 44:138–49. 10.1002/cmr.a.21339 26755895 PMC4706083

[28] Piccini D Monney P Sierro C Coppo S Bonanno G van Heeswijk RB. Respiratory self-navigated postcontrast whole-heart coronary MR angiography: initial experience in patients. Radiology. (2014) 270:378–86. 10.1148/radiol.13132045 24471387

[29] Cerqueira MD Weissman NJ Dilsizian V Jacobs AK Kaul S Laskey WK. Standardized myocardial segmentation and nomenclature for tomographic imaging of the heart. Circulation. (2002) 105:539–42. 10.1161/hc0402.102975 11815441

[30] Suther KR Hopp E Smevik B Fiane AE Lindberg HL Larsen S. Can visual analogue scale be used in radiologic subjective image quality assessment? Pediatr Radiol. (2018) 48:1567–75. 10.1007/s00247-018-4187-8 29974179 PMC6153875

[31] Wassmuth R Prothmann M Utz W Dieringer M von Knobelsdorff-Brenkenhoff F Greiser A. Variability and homogeneity of cardiovascular magnetic resonance myocardial T2-mapping in volunteers compared to patients with edema. J Cardiovasc Magn Reson. (2013) 15:27. 10.1186/1532-429X-15-27 23537111 PMC3627620

[32] Baeßler B Schaarschmidt F Stehning C Schnackenburg B Maintz D Bunck AC. A systematic evaluation of three different cardiac T2-mapping sequences at 1.5 and 3T in healthy volunteers. Eur J Radiol. (2015) 84:2161–70. 10.1016/j.ejrad.2015.08.002 26276731

[33] Wang X Kohler F Unterberg-Buchwald C Lotz J Frahm J Uecker M. Model-based myocardial T1 mapping with sparsity constraints using single-shot inversion-recovery radial FLASH cardiovascular magnetic resonance. J Cardiovasc Magn Reson. (2019) 21:60. 10.1186/s12968-019-0570-3 31533736 PMC6751613

[34] Granitz M Motloch LJ Granitz C Meissnitzer M Hitzl W Hergan K. Comparison of native myocardial T1 and T2 mapping at 1.5T and 3T in healthy volunteers: reference values and clinical implications. Wien Klin Wochenschr. (2019) 131:143–55. 10.1007/s00508-018-1411-3 30519737 PMC6459801

[35] Snel GJH van den Boomen M Hernandez LM Nguyen CT Sosnovik DE Velthuis BK. Cardiovascular magnetic resonance native T2 and T2^*^ quantitative values for cardiomyopathies and heart transplantations: a systematic review and meta-analysis. J Cardiovasc Magn Reson. (2020) 22:34. 10.1186/s12968-020-00646-8 32393281 PMC7212597

[36] Hamlin SA Henry TS Little BP Lerakis S Stillman AE. Mapping the future of cardiac MR imaging: case-based review of T1 and T2 mapping techniques. RadioGraphics. (2014) 34:1594–611. 10.1148/rg.346140030 25310419

[37] Galea N Rosato E Gigante A Borrazzo C Fiorelli A Barchetti G. Early myocardial damage and microvascular dysfunction in asymptomatic patients with systemic sclerosis: A cardiovascular magnetic resonance study with cold pressor test. PLoS ONE. (2020) 15:e0244282. 10.1371/journal.pone.0244282 33351821 PMC7755221

[38] Chareonthaitawee P Beanlands RS Chen W Dorbala S Miller EJ Murthy VL. Joint SNMMI–ASNC expert consensus document on the role of 18F-FDG PET/CT in cardiac sarcoid detection and therapy monitoring. J Nucl Cardiol. (2017) 24:1741–58. 10.1007/s12350-017-0978-9 28770463

[39] Darçot E Yerly J Colotti R Masci PG Chaptinel J Feliciano H. Accelerated and high-resolution cardiac T2 mapping through peripheral k-space sharing. Magn Reson Med. (2019) 81:220–33. 10.1002/mrm.27374 30058085

[40] Hamilton JI Jiang Y Chen Y Ma D Lo W-C Griswold M. MR fingerprinting for quantification of myocardial T1, T2, and M0. Magn Reson Med. (2017) 77:1446–58. 10.1002/mrm.26216 27038043 PMC5045735

[41] Milotta G Ginami G Bustin A Neji R Prieto C Botnar RM. 3D Whole-heart free-breathing qBOOST-T2 mapping. Magn Reson Med. (2020) 83:1673–87. 10.1002/mrm.28039 31631378 PMC7004111

[42] Bustin A Lima da Cruz G Jaubert O Lopez K Botnar RM Prieto C. High-dimensionality undersampled patch-based reconstruction (HD-PROST) for accelerated multi-contrast MRI. Magn Reson Med. (2019) 81:3705–19. 10.1002/mrm.27694 30834594 PMC6646908

