# Supplementary figures and images for: Respiratory Motion-Registered Isotropic Whole-Heart T2 Mapping in Patients With Acute Non-ischemic Myocardial Injury
Source: Front Cardiovasc Med. 2021 Sep 29;8:712383. doi: 10.3389/fcvm.2021.712383 (PMC8511642; doi:10.3389/fcvm.2021.712383)

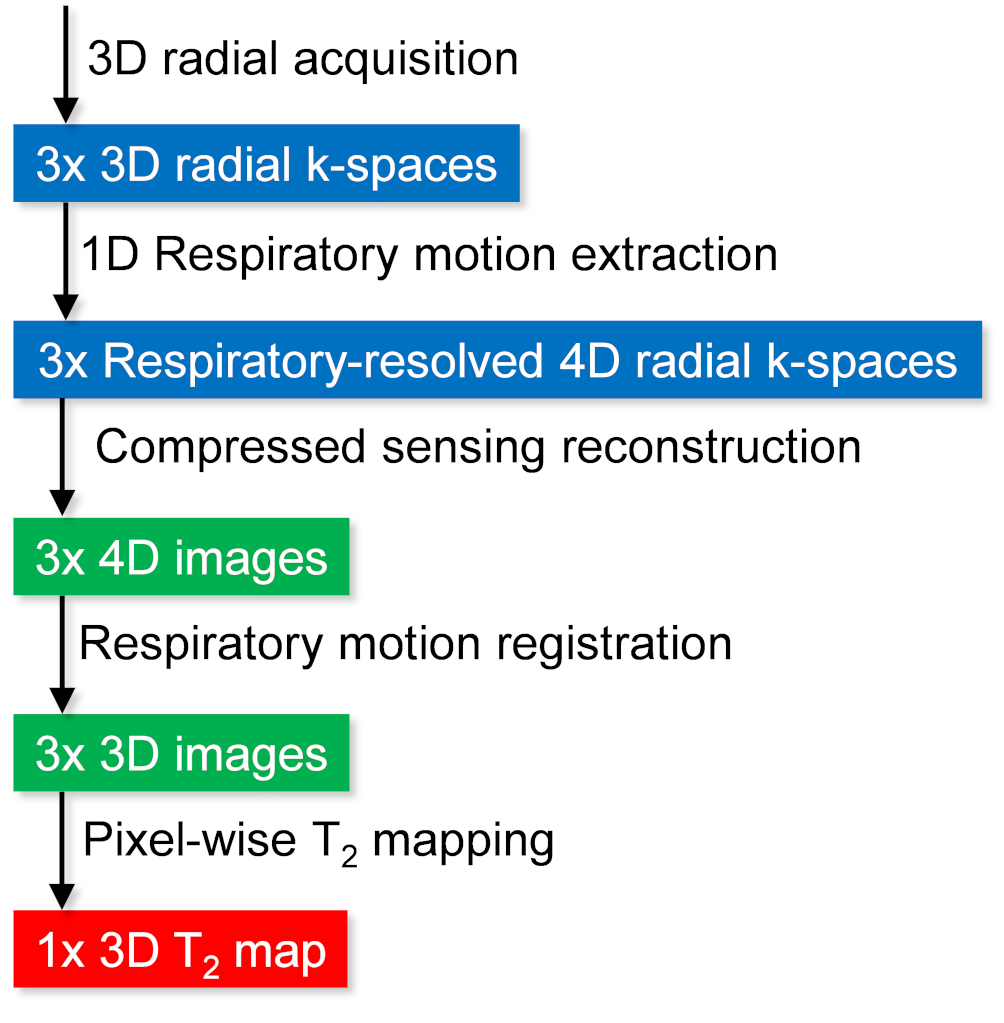

Supplement: Supplementary Figure 1 — A flowchart of the proposed 3D T2 mapping method. Data are indicated in colored boxes, while manipulations are mentioned as unboxed text. [file Image_1.TIFF]

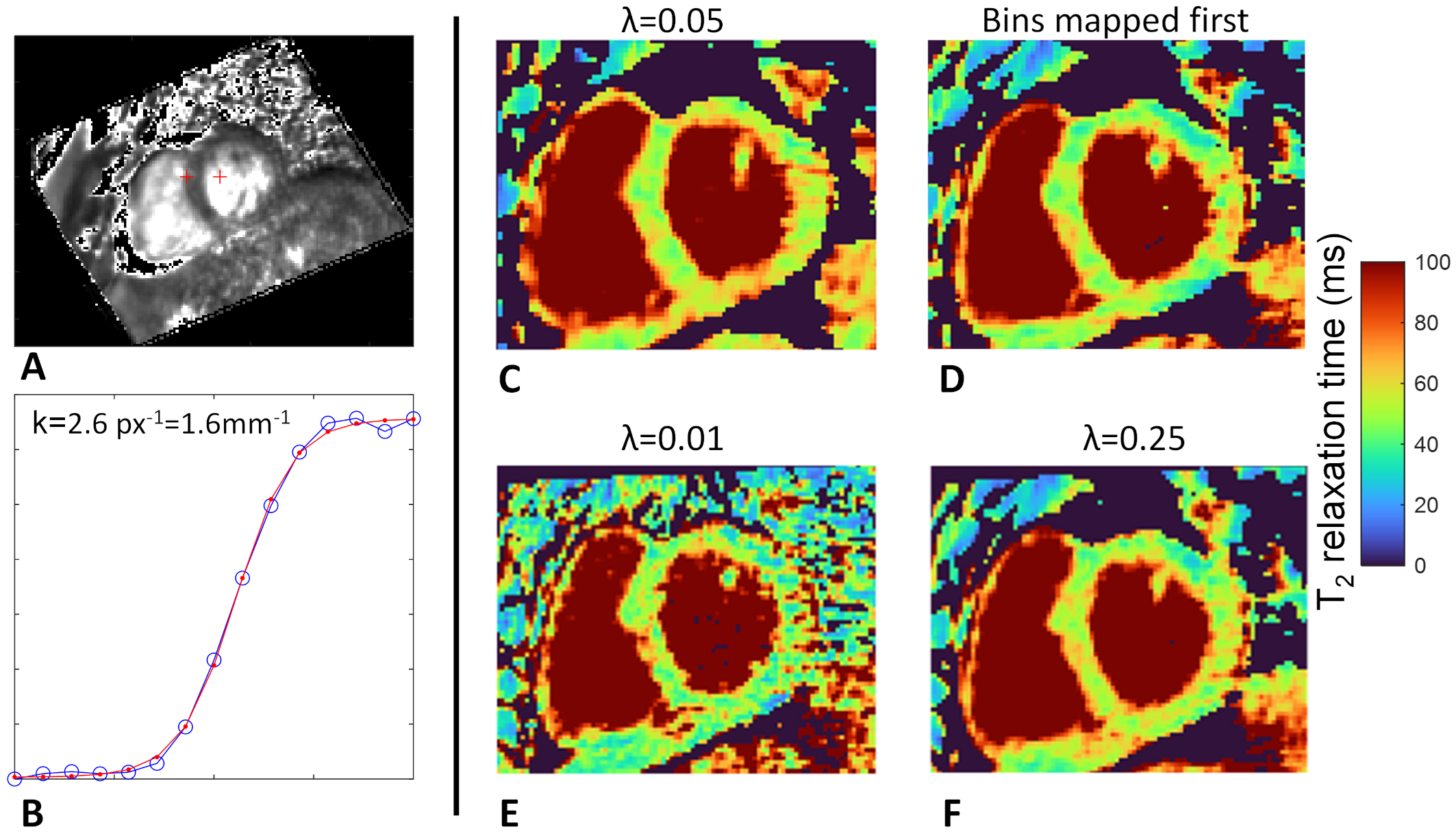

Supplement: Supplementary Figure 2 — Sharpness measurement and various reconstructions of a 3D T2 map. (A) A T2 map in grayscale with a display range from 0 to 300 ms to better visualize the blood pool, with red crosses indicating the start- and end-points of the assessed transition. Four horizontal pixel lines below it were also assessed. (B) Normalized T2 values in the pixel (blue circles) and the parametrized sigmoid fit (red curve). The sharpness of the transition was characterized by k = 2.6 px−1, which at the 1.6 mm spatial resolution translated to k = 1.6 mm−1. This value was averaged with those obtained from the lines below it. (C) The proposed 3D T2 mapping method in another patient. (D) An alternative reconstruction method in which the individual respiratory bins are all mapped first, and these bin maps are then averaged. While the papillary muscle appears to resolve better, the sharpness of the blood-myocardium interface remains constant. (E,F) The proposed reconstruction method, but with λ = 0.01 and 0.25. The blood-myocardium interfaces appear marginally sharper and more blurred, respectively, but this is balanced by respectively increased and decreased T2 variation in the myocardium. [file Image_2.TIFF]

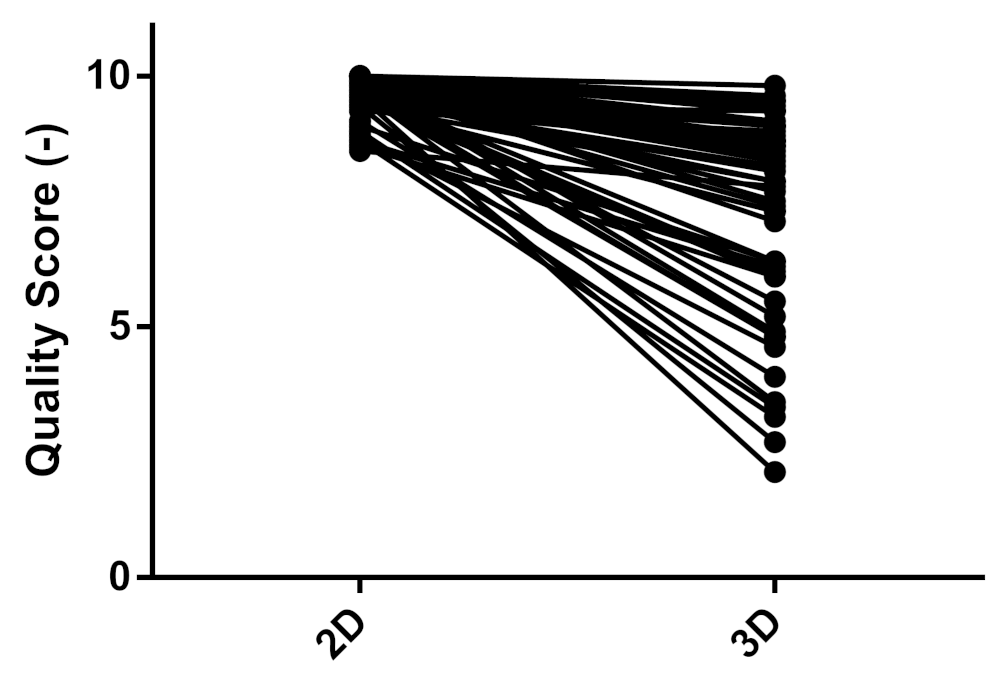

Supplement: Supplementary Figure 3 — A paired comparison of the visual quality scores as agreed by two experienced readers. The 3D T2 maps have a much higher variation in quality than the 2D T2 maps (7.2 ± 2.1 vs. 9.6 ± 0.4, P < 0.001), although it should be noted that the 2D T2 maps were immediately re-acquired during the scanning sessions if they were visually of unsatisfactory quality. [file Image_3.tif]

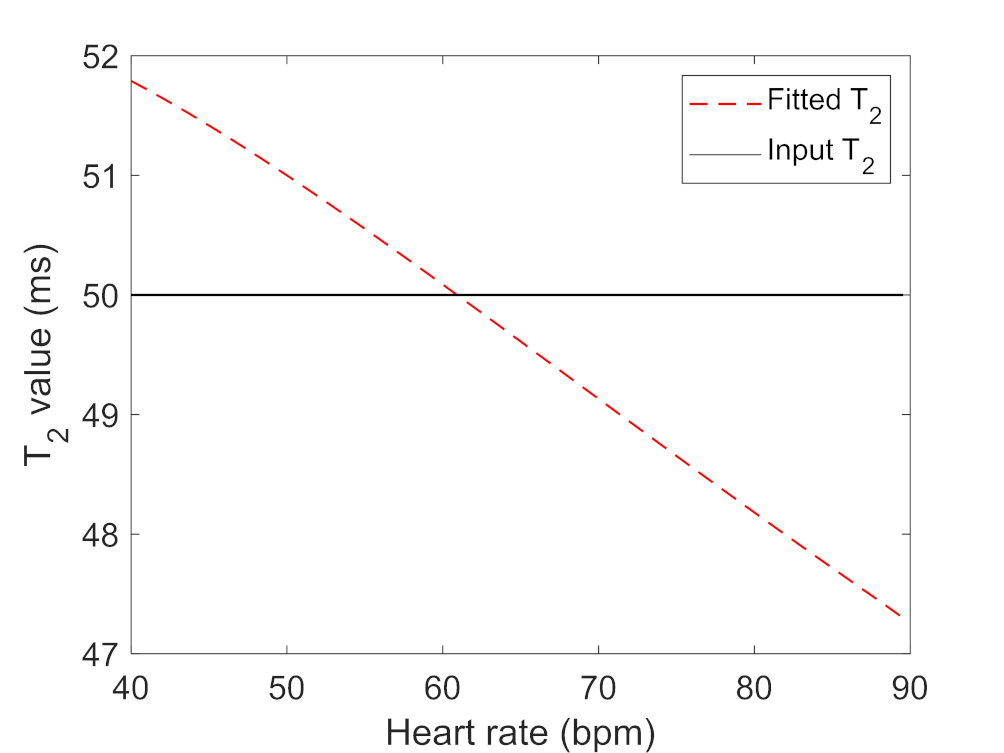

Supplement: Supplementary Figure 4 — A Bloch equation simulation of the influence of the patient's heart rate on the estimated T2 value. Since sampling occurs every other heartbeat, T1 relaxation will cause a gradual underestimation of the T2 value with the increase of the heart rate. [file Image_4.TIFF]
